# Global Measures of HIV Care Accessibility Across Urban, Suburban, and Rural Areas

**DOI:** 10.1007/s11524-025-01021-7

**Published:** 2025-12-08

**Authors:** Fabiana Cristina Dos Santos, Panta Apiruknapanond, Tongyao Wang, Carol Dawson-Rose, Claudia P. Valencia-Molina, Christine Horvat Davey, Solymar Solís Báez, Emilia Iwu, Motshedisi Sabone, Lufuno Makhado, J. Craig Phillips, Inge B. Corless, Sheila Shaibu, Wei-Ti Chen, Diane Santa Maria, Yvette P. Cuca, Rebecca Schnall

**Affiliations:** 1 Purdue University School of Nursing, Johnson Hall, 502 N. University Street, West Lafayette, IN 47907 USA; 2 Chulalongkorn University, St. Louis College, School of Nursing, Bangkok, Thailand; 3 The University of Hong Kong, School of Nursing, Pokfulam, Hong Kong; 4 Department of Community Health Systems, University of California, San Francisco (UCSF), School of Nursing, San Francisco, CA USA; 5 Universidad del Valle, Escuela de Enfermería, Facultad de Salud, Cali, Colombia; 6 Case Western Reserve University, Frances Payne Bolton School of Nursing, Cleveland, OH USA; 7 Auxilio Mutuo Hospital, San Juan, Puerto Rico; 8 School of Nursing, Center for Global Health, and Senior Technical Advisor, Institute of Human Virology, Rutgers University, Newark, NJ USA; 9 Retired Professor of Nursing, Gaborone, Botswana; 10 Department of Public Health, University of Venda, Limpopo Province, South Africa; 11 Faculty of Health Sciences, School of Nursing, University of Ottawa, Ottawa, Canada; 12 MGH Institute of Health Professions, Boston, MA USA; 13 Aga Khan University, School of Nursing, Nairobi, Kenya; 14 University of California, Los Angeles, Joe C. Wen School of Nursing, Los Angeles, CA USA; 15 University of Texas Health Science Center at Houston, Cizik School of Nursing, Houston, TX USA; 16 Columbia University, School of Nursing, New York, USA; 17 International Nursing Network for HIV Research, Ottawa, Canada

**Keywords:** HIV/AIDS, Global, Geographic location, Health disparities, Care accessibility

## Abstract

Despite advances in HIV treatment, disparities in healthcare access remain across low-income (LIC), middle-income (MIC), and high-income (HIC) countries, limiting access to clinical care, antiretroviral therapy, and viral load testing. This study developed the HIV Care Access Index (HIV-CAI) to measure and compare HIV care service access across countries and within geographic regions and examined associations with economic and health indicators. A cross-sectional survey was conducted between August 2021 and June 2023 across nine countries: Botswana, China, Colombia, Kenya, Nigeria, Puerto Rico, South Africa, Thailand, and the United States. Participants were adults living with HIV recruited from urban, suburban, and rural settings through HIV service organizations, sexual and gender minority centers, and resource-limited communities. The HIV-CAI was scored from 0 to 1 (worst to best access) across three domains: Access to HIV Clinical Care and Providers, Access to HIV Medication, and Access to Viral Load Testing. Among 1,598 participants, Botswana demonstrated the highest HIV care access (0.93), followed by Thailand (0.91) and Kenya (0.90), while Nigeria showed the lowest access (0.64). Access to HIV medication was relatively consistent across regions, whereas access to clinical care and providers was limited, particularly in rural areas. Countries with higher HIV burden, incidence, and prevalence were associated with better care access. The HIV-CAI reveals disparities in HIV care access across geographic areas. Future interventions should prioritize improving equitable access, particularly in rural areas and countries with lower overall access scores.

**Supplementary Information:**

The online version contains supplementary material available at 10.1007/s11524-025-01021-7.

## Introduction

HIV remains a global health threat, with 1.5 million new infections and 650,000 HIV-related deaths worldwide in 2021 [1]. In response, evidence-based initiatives such as the Joint United Nations Program on HIV/AIDS (UNAIDS) have intensified efforts to prioritize HIV prevention and care and end the HIV epidemic by 2030 [2]. Central to this goal is ensuring that individuals diagnosed with HIV initiate antiretroviral therapy (ART) immediately and achieve viral suppression [3]. Viral suppression refers to reducing viral loads in the blood to undetectable amounts through effective ART, which not only prevents disease progression but also eliminates the risk of HIV transmission. Achievement of these targets by 2030 is estimated to result in a 95% reduction in HIV incidence and HIV-related mortality worldwide [2, 4]. However, HIV care access remains a critical issue globally, affecting millions of people with HIV (PWH) [5, 6]. HIV care is a comprehensive approach that emphasizes efficient and early linkage to care, expanded HIV testing, access to antiretroviral therapy with adherence support, strategies to promote patient retention, and integration of social services to address psychosocial needs. It also requires interventions targeting social determinants of health, policies that ensure equitable access to healthcare, and the delivery of culturally competent care. Collectively, these efforts are crucial to achieving effective viral suppression, improving quality of life, and preventing HIV transmission [7, 8].

Despite significant advances in ART and efforts to improve healthcare delivery [9–12], many individuals still face substantial disparities in access to HIV care worldwide [13–15], particularly in regions with the largest HIV burden, such as Africa, which cumulatively accounts for 57% of all new HIV infections [6].

The discrepancy in HIV care access between low-income (LIC), medium-income (MIC), and high-income (HIC) countries remains [5, 15]. This gap is aggravated by a deficiency of educational resources, limited healthcare infrastructure, and financial instability, all of which are structural factors that the COVID-19 pandemic has further intensified [16–18]. As a result, PWH in LIC, MIC, and HIC countries [12, 13, 15, 19] often face challenges in accessing specialized clinics and providers, maintaining consistent ART, receiving regular viral load testing, and engaging with other critical components of HIV care. Geographic disparities exacerbate these problems, with rural populations frequently experiencing worse health outcomes than those in urban areas. For instance, analyses of the Centers for Disease Control and Prevention’s National HIV Surveillance System in the United States [20] have shown that rural residents have lower rates of care retention and viral suppression compared to those in urban areas. Similarly, a study [21] conducted in Nigeria demonstrated these disparities, showing lower rates of care retention, viral suppression, and HIV testing in non-metropolitan areas, highlighting the urgent need for targeted interventions to address geographic inequities in HIV care delivery. While these studies underscore the magnitude of disparities, the measurement approaches might be fragmented, focusing on single outcomes (e.g., ART uptake or viral suppression) or limited to specific national or regional contexts.

Researchers and healthcare providers have faced challenges in effectively measuring the factors associated with HIV care and accessibility across countries. Current frameworks, such as the HIV Care Continuum, have provided meaningful insights into the progression from HIV diagnosis to viral suppression [22]. This internationally recognized framework represents HIV care as a process from testing to engagement in clinics, ART adherence, and, ultimately, viral suppression [23]. This framework guided our analysis of three key components of HIV care accessibility: HIV clinical care and HIV care providers, HIV medication, and viral load testing.

This analysis aimed to measure and compare HIV care accessibility across different sites and develop the HIV Care Access Index (HIV-CAI). It incorporates 1) Access to HIV Clinical Care and HIV Care Providers, 2) Access to HIV Medication, and 3) Access to Viral Load Testing.

HIV-CAI provides a comprehensive metric that captures accessibility across multiple domains of care, diverse geographic contexts (urban, suburban, and rural), and economic settings (LIC, MIC, and HIC countries). Whereas UNAIDS’ 95–95-95 targets provide global benchmarks for diagnosis, treatment, and viral suppression, the HIV-CAI complements these indicators by assessing HIV care services availability across different geographic areas and economic contexts. The HIV-CAI advances current knowledge by enabling cross-national and cross-regional comparisons of care accessibility, identifying potential structural barriers, and providing a foundation for targeted, context-specific interventions to reduce inequities in HIV care worldwide. We also aim to provide a tool for assessing and comparing HIV care accessibility globally.

## Method

### Data Sources

This secondary analysis utilized cross-sectional survey data collected between August 2021 and June 2023 as part of the International Nursing Network for HIV Research Study VIII [18]. The study aimed to assess the impact of the COVID-19 pandemic on PWH in 10 countries. The dataset included HIV care variables, such as access to specialized clinics and healthcare providers, initiation of ART, adherence to treatment regimens, and access to HIV viral load testing [24]. This global survey aimed to understand the impact of the COVID-19 epidemic on PWH across the globe. Detailed information about the survey can be found in the study protocol [18].

Eligible participants were adults living with HIV in urban, suburban, and rural areas and who met their local age of consent (21 years in San Juan, Puerto Rico; 18 years at other sites). Participants were recruited from diverse settings, including HIV service organizations, sexual and gender minority centers, and resource-limited communities. The study sites included 10 countries: Botswana (*n* = 100), Canada (*n* = 3), China (*n* = 270), Colombia (*n* = 100), Kenya (*n* = 112), Nigeria (*n* = 119), Puerto Rico (*n* = 23), South Africa (*n* = 285), Thailand (*n* = 104), and the United States (*n* = 485), resulting in a sample size of 1,601 persons who completed the survey. The location where participants live was assessed with a single question. Participants were asked to select one of the following options: ‘Do you live in an area that is (1) Urban (city), (2) Suburban (around a city), or (3) Rural?’.

Geographic areas with fewer than 10 observations were excluded from the analysis to ensure statistical reliability and representativeness of the data. To capture variation across diverse geographic and economic contexts, the enrollment areas from the nine participating countries were grouped according to the Global Burden of Disease (GBD) super-region scheme [25]. These regions included High-income North American countries (United States), the Caribbean (Puerto Rico), Central Latin American (Colombia), Southeast Asia (Thailand), East Asia (China), Southern Sub-Saharan Africa (Botswana and South Africa), Eastern Sub-Saharan Africa (Kenya), and Western Sub-Saharan Africa (Nigeria) [25]. Although Puerto Rico is a United States territory, we analyzed its data separately from other United States jurisdictions due to its distinctive epidemiological profile and highest rates of HIV prevalence and incidence [26]. The reason for using the GBD super-region scheme was to ensure that our analysis reflects diverse global contexts and allows for meaningful comparisons of HIV care accessibility across regions that differ in epidemiological patterns, health system capacity, and socioeconomic conditions. By including at least one country from each of these super-regions, the HIV-CAI offers a standardized tool to assess and compare HIV care access not only within countries but also across broader global regions.

In addition to the survey data, we used public data from the World Development Indicators (year 2020)[27] to describe relevant country-level economic and health factors (Supplement, eTable 1). Specifically, we included measures of financial capacity (Gross Domestic Product [GDP], Poverty Headcount Ratio, and Prevalence of Severe Food Insecurity), health system investment (Current Health Expenditure as a percentage of GDP and per capita in PPP-adjusted dollars), and HIV-specific outcomes (Incidence of HIV, Prevalence of HIV among individuals ages 15–49, and ART coverage among people living with HIV), as well as population size (Total Population). These indicators were selected because they provide comparable metrics across countries, allowing us to contextualize survey findings and explore how macro-level economic and health system factors may contribute to HIV care accessibility.

### Development of the HIV Care Access Index

#### Selection of Variables

HIV care refers to ensuring that all individuals have adequate access to treatment, beginning with diagnosis and continuing through linkage to care, initiation of antiretroviral therapy, adherence to the treatment regimen, and ultimately achieving viral suppression to an undetectable level [22]. The HIV Care Continuum Framework [22] posits that there are three main domains to HIV care access: 1) Access to HIV Clinical Care and HIV Care Providers, 2) Access to HIV Medication, and 3) Access to Viral Load Testing. By dividing HIV care access into these three main domains, our analysis focuses on each aspect of care and investigates how equitably HIV care access is distributed across different geographic areas. HIV care access was measured using a total of 12 variables collected in the survey, of which five represent access to HIV clinical care and HIV care providers, five other variables describe access to HIV medication, and two characterize access to viral load testing (Supplement, eTable 2). A brief description of each domain appears below.

- Domain 1: Access to HIV Clinical Care and HIV Care Providers (*n* = 5): Captures the accessibility and continuity of HIV care through access to HIV healthcare services, including provider visits, clinical consultations, prescriptions for medication, and the availability of pharmacy services.
- Domain 2: Access to HIV Medication (*n* = 5): Evaluates accessibility of HIV care by examining several aspects of antiretroviral medication access, including overall medication usage, daily antiretroviral adherence to prescribed regimens, and access to medications over the past 3 and 30 days.
- Domain 3: Access to Viral Load Testing (*n* = 2): Categorizes the frequency and accessibility of viral load monitoring among PWH to manage and adjust treatment regimens. For instance, self-reported questions assess whether participants have had access to the most recent viral load testing or other necessary laboratory exams.

#### Data Transformation and Index Calculation

The original survey data consisted of 12 categorical variables that were transformed into 17 normalized variables (Supplement, eTable 3). This normalized dataset included 14 binary variables and three continuous variables. All variables range between 0 and 1, where ‘1’ represents the best access to HIV clinical care and HIV care provider, HIV medication, and viral load testing, and ‘0’ the worst. This normalization was performed to ensure comparable measurement scales across all dimensions of HIV care access.

The HIV-CAI was constructed using a two-step process: 1) Individual-level aggregation and 2) Country-level aggregation. In the first step, we calculate the average of each one of the 17 normalized variables across all individuals within the same country, resulting in 17 scores between 0 and 1. The second step is then to take the average across the 17 scores for each country, resulting in the country-level HIV Care Access Index.

The HIV-CAI represents the country-level mean of all scores, providing an overall assessment of HIV care access across the population studied. This Index ranges from ‘0’ to ‘1,’ where ‘0’ represents the worst HIV care access, and a value of ‘1’ represents the best care access. Equation (1) states the index calculation, where ${\overline{X} }_{k}$ represents the *k* score variable. This approach ensures equal weighting of all variables in the index while accounting for potential differences in sample sizes across countries, resulting in a standardized measure that facilitates cross-country comparisons.

1 $$HIV\;Care\;Access\;Index= \frac{\sum_{k=1}^{17}{\overline{X} }_{k}}{17}$$

#### Index Application

In addition to the HIV-CAI, we also constructed sub variations of the index by measuring care access in rural, suburban, and urban areas across different countries, allowing us to identify disparities in HIV care access within countries, and to provide insights into the continuity and quality of care service delivery in areas of the country with different levels of urbanization.

### Data Analysis

Descriptive statistics, including absolute numbers and percentages, were used to describe the sample. The HIV Care Access Index (HIV-CAI) is calculated from the averages of each variable across all individuals within the same country. To complement this analysis, we also construct the equivalent of the index using different percentiles of the distribution, such as the 25th, 50th, and 75th percentiles (See eTable 4). To explore the association between the HIV-CAI and country-level economic and health-related data from the World Development Indicators, the Pearson correlation coefficients were calculated. This analysis examined associations between the HIV-CAI and country-level indicators from the World Development Indicators data [27]. This approach enabled us to explore how the HIV-CAI relates to broader economic and health contexts across countries, providing insights into potential factors that may influence access to HIV care services. While the correlation analysis provides a summary of the strength and direction of linear relationships between economic and health variables, it may not capture the entire nature of these associations. A scatterplot matrix was generated to visualize the relationships between variables (eFigure 1). This visualization complemented the correlation analysis by revealing potential non-linear patterns and outliers that might influence the observed relationships. Statistical analyses were conducted using RStudio software version 4.2.1 (R Foundation).

## Results

A total of 1,598 study participants were included in this analysis. The largest cohorts were from the United States (*n* = 485, 30.4%) and South Africa (*n* = 285, 17.8%), followed by China (*n* = 270, 16.9%). Ages varied across countries, with the United States reporting the highest mean age (51.6 years, SD = 13.3) and Nigeria the lowest (35.0 years, SD = 10.3). The majority of participants resided in urban areas (*n* = 941, 59%), followed by rural (*n* = 366, 23%) and suburban areas (*n* = 291, 18%) (Table 1).

**Table 1:** Characteristics of the Participants at Baseline by Country (*n* = 1,598)

n (%)	Botswana (*n* = 100)	China (*n* = 270)	Colombia (*n* = 100)	Kenya (*n* = 112)	Nigeria (*n* = 119)	Puerto Rico (*n* = 23)	South Africa (*n* = 285)	Thailand (*n* = 104)	United States (*n* = 485)
Age Mean (SD)	43.4 (10.0)	37.0 (11.8)	41.3 (14.7)	38.2 (10.0)	35.0 (10.3)	45.6 (12.3)	45.3 (12.7)	44.6 (12.7)	51.6 (13.3)
Sex at birth									
Female Male	55 (55) 45 (45)	19 (7) 251 (93)	43 (43) 57 (57)	86 (77) 26 (23)	65 (55) 54 (45)	6 (26) 17 (74)	171 (60) 114 (40)	45 (43) 59 (57)	145 (30) 340 (70)
Gender Identity									
Female Male Transgender F Transgender M Genderqueer Other	54 (54) 45 (45) 0 1 (1) 0 0	19 (7) 240 (89) 0 3 (1) 3 (1) 5 (2)	43 (43) 46 (46) 7 (7) 1 (1) 2 (2) 1 (1)	83 (74) 26 (23) 0 0 1 (1) 2 (2)	60 (50) 50 (42) 1 (1) 0 1 (1) 7 (6)	6 (26) 13 (57) 3 (13) 0 0 1 (4)	171 (60) 114 (40) 0 0 0 0	45 (43) 46 (44) 0 0 13 (13) 0	145 (30) 320 (66) 10 (2) 5 (1) 5 (1) 0
Race									
Black White Asian American Indian Hawaiian Multiracial Other	100 (100) 0 0 0 0 0 0	0 0 270 (100) 0 0 0 0	17 (17) 18 (18) 0 1 (1) 0 52 (52) 12 (12)	110 (98) 0 0 0 0 0 2 (2)	61 (51) 0 2 (2) 0 0 15 (13) 41 (34)	7 (30) 4 (18) 0 0 0 12 (52) 0	285 (100) 0 0 0 0 0 0	0 0 104 (100) 0 0 0 0	252 (52) 141 (29) 5 (1) 0 0 48 (10) 39 (8)
Hispanic (yes)	0	0	96 (96)	2 (2)	10 (8)	21 (91)	0	0	97 (20)
Higher education	20 (20)	205 (76)	44 (44)	21 (19)	52 (44)	14 (61)	37 (13)	36 (35)	306 (63)
Areas									
Urban Suburban Rural	60 (60) 20 (20) 20 (20)	248 (92) 19 (7) 3 (1)	88 (88) 2 (2) 10 (10)	35 (31) 73 (65) 4 (4)	80 (67) 16 (14) 23 (19)	20 (87) 2 (9) 1 (4)	3 (1) 0 282 (99)	33 (32) 67 (64) 4 (4)	374 (77) 92 (19) 19 (4)

### HIV Care Access across Countries

**Fig. 1:**
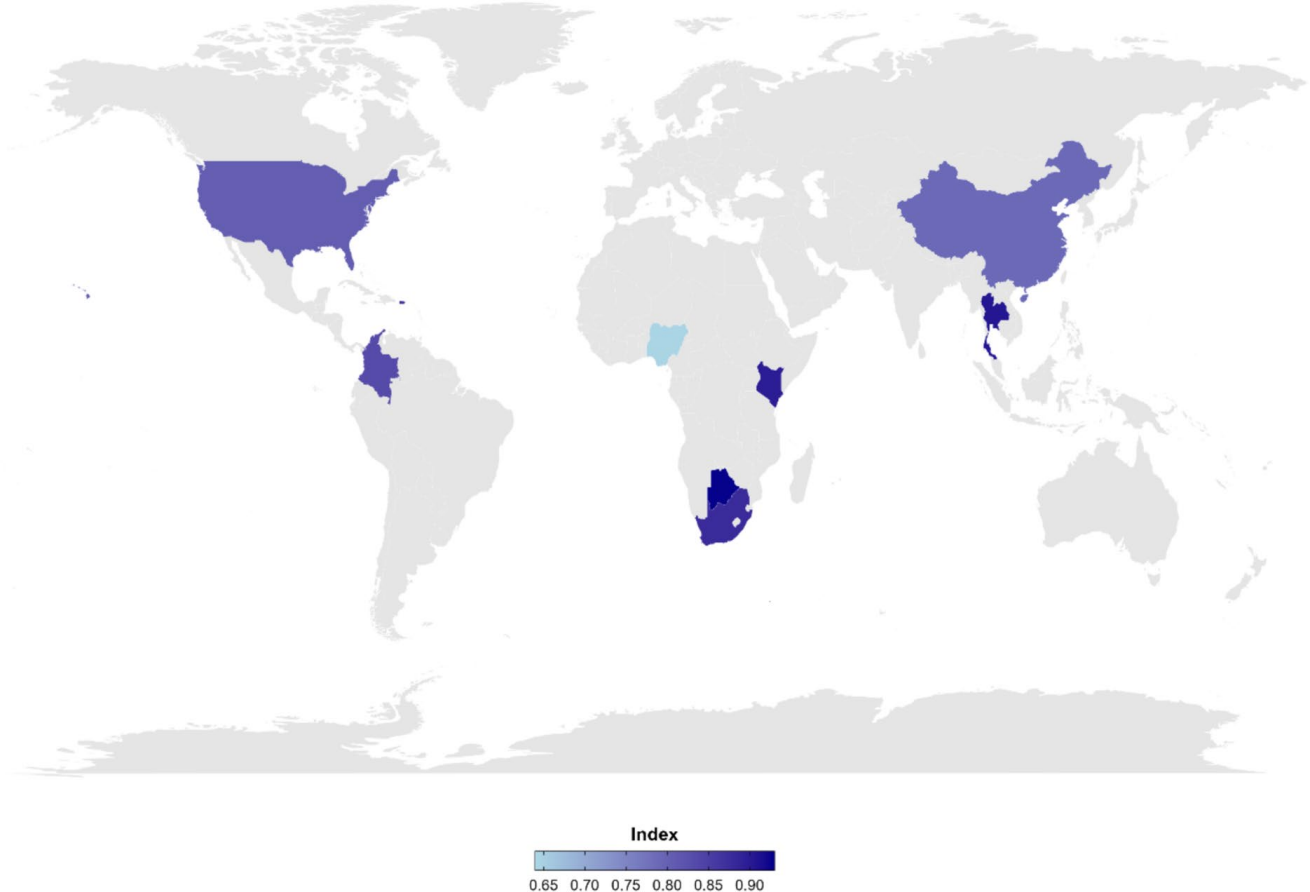
Global HIV Care Accessibility Index: Illustration of HIV care access across countries. The blue gradient variation indicates access levels to overall HIV care. The dark blue represents the higher access to HIV care in that area

The overall HIV-CAI revealed varying levels of access to HIV care services across the nine countries, with results illustrated using a gradient of blue (Fig. 1). Botswana had the highest overall score (Index = 0.93) compared to other studied countries, indicating better HIV care access, followed by Thailand (Index = 0.91) and Kenya (Index = 0.90) (Table 2). While Botswana has the highest overall HIV-CAI among the countries, Nigeria has the lowest Index score of 0.64, with the scale ranging from 0 (worst access) to 1 (better access). This Index score suggests that there are still gaps in achieving optimal HIV care. This Index reflects the necessity of ongoing evaluation and adjustment of HIV care programs to meet PWH's needs.

**Table 2:** HIV Care Access Index Across Countries

Country	Sample Size (*n* = 1,598)	Overall HIV Care Access Index	HIV Care Access Index by Domain		
			1. HIV Clinical Care and HIV Care Providers	2. HIV Medication	3. Viral Load Testing
Botswana	100	0.93	0.86	0.98	0.93
Colombia	100	0.83	0.77	0.89	0.82
China	270	0.79	0.65	0.87	0.85
Kenya	112	0.90	0.84	0.96	0.85
Nigeria	119	0.64	0.58	0.72	0.58
Puerto Rico	23	0.88	0.78	0.94	0.90
South Africa	285	0.88	0.83	0.95	0.79
Thailand	104	0.91	0.82	0.97	0.91
United States	485	0.81	0.75	0.88	0.75

Noteworthy differences were observed within specific domains of HIV care access. Botswana demonstrated consistently high Index scores across all three domains, leading in access to HIV clinical care and HIV care providers (Index = 0.86), access to HIV medication (Index = 0.98), and access to viral load testing (Index = 0.93) among the countries. Kenya achieved the second-highest score for access to HIV clinical care and HIV care providers (Index = 0.84), and Thailand showed a high score for access to HIV medication (Index = 0.97).

In contrast, Nigeria showed the worst access across all domains with the lowest scores for access to HIV clinical care and providers (Index = 0.58), viral load testing (Index = 0.58), and HIV medication (Index = 0.72). China demonstrated the second-lowest score for HIV clinical care and HIV care providers (Index = 0.65), while the United States, despite having moderate overall access, showed the lowest index scores in access to HIV clinical care and HIV care providers (Index = 0.75) and viral load testing (Index = 0.75). These findings indicate the need for targeted interventions to address domain-specific gaps in HIV care services across different countries (Table 2).

### Correlation of HIV-CAI and Country-Level Variables

Table 3 presents associations between the HIV Care Access Index, economic, and health-related factors across countries studied. We found that HIV-CAI was negatively correlated with total population (r = −0.37), suggesting that in more populous countries, access disparities may persist, potentially due to geographic inequities or resource fragmentation. Among the health-related factors of the countries studied, we found that the HIV-CAI index was positively correlated with HIV prevalence (r = 0.44), HIV incidence (r = 0.38), and ART coverage (r = 0.19), suggesting that care access tends to be better in countries with higher HIV burden.

**Table 3:** Economic and Health-Related Determinants Associated with HIV Care Access

	Health Expenditure^a^	HIV Incidence^b^	Total Population^c^	HIV Prevalence^d^	Food Insecurity^e^	Poverty^f^	ART Coverage^g^	Health Expenditure^h^	GDP^i^	Index^j^
Health Expenditure^a^	1									
HIV Incidence^b^	0.04	1								
Total Population^c^	0.09	−0.31	1							
HIV Prevalence^d^	0.03	0.99	−0.32	1						
Food Insecurity^e^	−0.25	0.46	−0.32	0.51	1					
Poverty^f^	0.18	−0.45	0.14	−0.42	0.01	1				
ART Coverage^g^	−0.23	0.49	−0.56	0.51	0.62	0.04	1			
Health Expenditure^h^	0.91	−0.19	0.10	−0.19	−0.31	0.09	−0.47	1		
GDP^i^	0.68	−0.35	0.69	−0.36	−0.43	0.15	−0.73	0.79	1	
Index^j^	−0.06	0.38	−0.37	0.44	0.03	0.17	0.19	−0.12	−0.28	1

a. Current health expenditure (% of GDP) [SH.XPD.CHEX.GD.ZS]. b. Incidence of HIV, all (per 1,000 uninfected population) [SH.HIV.INCD.TL.P3]. c. Population, total [SP.POP.TOTL]. d. Prevalence of HIV, total (% of population ages 15–49) [SH.DYN.AIDS. ZS]. e. Prevalence of severe food insecurity in the population (%) [SN.ITK.SVFI. ZS]. f. Poverty headcount ratio at societal poverty line (% of population) [SI.POV.SOPO]. g. Antiretroviral therapy coverage (% of people living with HIV) [SH.HIV.ARTC. ZS]. h. Current health expenditure per capita, PPP (current international $) [SH.XPD.CHEX.PP.CD]. i. GDP (constant 2015 US$ [NY.GDP.MKTP. KD]. j. HIV Care Access Index. Data from the database World Development Indicators [Last Updated: 03/28/2024]

These correlations suggest that HIV care access is influenced by both epidemic burden and economic capacity through complex, non-linear relationships. While higher HIV prevalence and incidence were linked to improved access, likely due to prioritization of HIV programs and allocated resources, more populous countries did not necessarily translate into better access. These findings underscore the importance of examining beyond aggregate economic indicators to comprehend HIV care accessibility, reinforcing the value of the HIV-CAI as a complementary tool to global benchmarks.

### HIV Care Access across Rural, Suburban, and Urban Areas

Table 4 presents disparities in access to HIV care across urban, suburban, and rural areas. Overall, substantial variation was observed within and across countries. Overall, HIV care access indices ranged from 0.62 (Nigeria, urban) to 0.96 (Botswana, rural), indicating variation within and across countries.

**Table 4:** Disparities in HIV Care Access across Urban, Suburban, and Rural Areas

Country	Sample Size	Area	Overall HIV Care Access Index	Access to HIV Clinical Care and HIV Care Providers	Access to HIV Medications	Access to Viral Load Testing
Botswana	60	Urban	0.92	0.86	0.97	0.91
	20	Suburban	0.92	0.85	0.97	0.92
	20	Rural	0.96	0.88	1	1
China	248	Urban	0.79	0.65	0.87	0.86
	18	Suburban	0.74	0.60	0.82	0.80
Colombia	88	Urban	0.85	0.78	0.91	0.83
	10	Rural	0.71	0.68	0.72	0.77
Kenya	35	Urban	0.89	0.84	0.96	0.81
	73	Suburban	0.90	0.85	0.96	0.85
Nigeria	79	Urban	0.62	0.54	0.70	0.55
	16	Suburban	0.80	0.78	0.82	0.77
	21	Rural	0.66	0.59	0.74	0.57
Puerto Rico	20	Urban	0.86	0.77	0.93	0.88
South Africa	282	Rural	0.88	0.83	0.95	0.79
Thailand	32	Urban	0.91	0.82	0.97	0.94
	67	Suburban	0.90	0.82	0.96	0.90
United States	375	Urban	0.8	0.74	0.87	0.75
	86	Suburban	0.84	0.76	0.91	0.80
	17	Rural	0.82	0.86	0.84	0.71

Note that some regions were excluded from the analysis because our criteria required a minimum of 10 observations

In Botswana (*n* = 100), HIV care access was uniformly high, in urban (0.92; *n* = 60) and suburban (0.92; *n* = 20) areas, with rural areas showing slightly higher access (0.96; *n* = 20). Similarly, Thailand (*n* = 99) and Kenya (*n* = 108) demonstrated consistently high access, with differences between urban and suburban areas (Thailand: 0.91 vs. 0.90; Kenya: 0.89 vs. 0.90). South Africa (*n* = 282) also reported high levels of access in rural populations (0.88), although urban and suburban data were not available for comparison. By contrast, several countries exhibited notable geographic disparities. In Colombia (*n* = 98), participants who reported living in urban areas presented higher access (0.85; *n* = 88) compared with rural participants (0.71; *n* = 10). In China (*n* = 266), urban areas demonstrated better access (0.79; *n* = 248) compared to suburban areas (0.74; *n* = 18). Conversely, Nigeria (*n* = 116) showed higher access in suburban areas (0.80; *n* = 16) compared with rural areas (0.66; *n* = 21) and, especially, urban areas (0.62; *n* = 79).

In the United States (*n* = 478), disparities were more moderated. Suburban areas demonstrated the highest access (0.84; *n* = 86), followed by rural areas (0.82; *n* = 17) and urban areas (0.80; *n* = 375). Puerto Rico (*n* = 20), where only urban data was available, reported high HIV care access (0.86). Countries such as Botswana, Thailand, Kenya, and South Africa demonstrated better access to HIV care across all available geographic areas, while Colombia (urban vs. rural) and Nigeria (suburban vs. urban) were observed as subpopulations with comparatively lower access to HIV care. However, these findings should be interpreted with caution, given the unequal and small sample sizes across countries and geographic areas.

### Access to HIV Clinical Care and HIV Care Providers

Table 4 summarizes disparities in access to HIV clinical care and HIV care providers across urban, suburban, and rural areas. HIV-CAI scores ranged from 0.54 (Nigeria, urban) to 0.88 (Botswana, rural), suggesting wide variability across both countries and areas.

In Botswana, access was uniformly high across all areas, urban (0.86; *n* = 60), suburban (0.85; *n* = 20), and rural participants (0.88; *n* = 20). Thailand and Kenya also demonstrated consistently high access across urban and suburban areas (Thailand: 0.82 vs. 0.82; Kenya: 0.84 vs. 0.85). Similarly, South Africa showed better access among rural areas (0.83), although data for urban and suburban areas were not collected.

Other countries, however, demonstrated disparities. For instance, in Colombia (*n* = 98), urban areas showed better access (0.78; *n* = 88) compared with rural areas (0.68; *n* = 10), followed by China, where urban areas displayed better access (0.65; *n* = 248) compared with suburban areas (0.60; *n* = 18). In Nigeria, suburban areas showed better HIV clinical care access (0.78; *n* = 16) compared with rural (0.59; *n* = 21) and urban areas (0.54; *n* = 79).

### Access to HIV Medication

Table 4 presents access to HIV medications across urban, suburban, and rural areas. Access to medication was generally high across most countries, with scores ranging from 0.70 (Nigeria, urban) to 1.00 (Botswana, rural). In Botswana, access to medications was nearly uniform, urban (0.97; *n* = 60), suburban (0.97; *n* = 20), and rural areas (1.00; *n* = 20). Thailand and Kenya demonstrated similarly high access to medication in urban and suburban areas (Thailand: 0.97 vs. 0.96; Kenya: 0.96 vs. 0.96), while South Africa (*n* = 282) showed better access among rural populations (0.95).

Despite access to HIV medication, notable geographic disparities emerged in several countries. In Colombia, for instance, urban areas showed better access (0.91; *n* = 88), whereas rural (0.72; *n* = 10) demonstrated lower access. Nigeria also displayed within-country disparities. Suburban areas showed higher medication access (0.82; *n* = 16) compared with rural areas (0.74; *n* = 21) and especially urban areas (0.70; *n* = 79).

In the United States, suburban areas showed the highest access (0.91; *n* = 86), followed by urban (0.87; *n* = 375) and rural areas (0.84; *n* = 17). Puerto Rico (*n* = 20), where only urban areas' data were observable in our data set, reported high medication access (0.93). As with previous analyses, results should be interpreted cautiously due to the small sample sizes across areas.

### Access to Viral Load Testing

Table 4 summarizes disparities in access to viral load testing across urban, suburban, and rural settings. Viral load testing access scores ranged from 0.55 (Nigeria, urban) to 1.00 (Botswana, rural).

In Botswana, access to viral load testing was uniform, with urban (0.91; *n* = 60) and suburban (0.92; *n* = 20) areas reporting high access, while rural areas showed full access for our sample (1.00; *n* = 20). Thailand also demonstrated better access across urban and suburban areas (0.94 vs. 0.90). In Kenya, viral load testing access was somewhat lower but still relatively high, in suburban areas (0.85; *n* = 73) and urban areas (0.81; *n* = 35).

In Nigeria, suburban areas showed the highest access (0.77; *n* = 16), compared with rural areas (0.57; *n* = 21) and, especially, urban areas (0.55; *n* = 79). The urban–suburban difference represented an important gap observed across all countries.

In the United States, suburban participants reported the highest access (0.80; *n* = 86), followed by urban (0.75; *n* = 375) and rural participants (0.71; *n* = 17). Puerto Rico (*n* = 20), where only urban data was available, reported high access (0.88).

## Discussion

This study introduces HIV-CAI as a novel tool for quantifying HIV care accessibility and comparing access to care services across nine countries while exploring differences between rural, urban, and suburban areas. Our results demonstrated that Botswana had the highest HIV-CAI scores, reflecting its long-standing investment in HIV care infrastructure, national ART programs, and integration of HIV services into routine clinical care [28, 29]. These findings corroborate a prior study that used data from the Fifth Botswana AIDS Impact Survey (BAIS V) to measure progress towards these treatment targets, resulting in high rates of viral suppression and progress toward the UNAIDS 95–ID="EN5">95-95 targets [29]. Similarly, Thailand ranked among the top countries, consistent with its history of leveraging health initiatives to expand HIV testing and treatment programs, which have contributed to sustained improvements in access to care [30].

In contrast, Nigeria reported the lowest HIV-CAI, particularly for clinical care and viral load testing accessibility. This finding aligns with prior studies that have documented persistent gaps in health system capacity, supply chain instability, and inadequate laboratory infrastructure [31, 32]. Nigeria remains a high-burden country, with about 1.9 million people living with HIV, and is one of the top five countries that account for the highest number of HIV-positive people in the world [32]. Over the last decade, Nigeria has made measurable progress in controlling its HIV epidemic, including a decrease from 4.1% in 2010 to 1.4% in 2019 in HIV prevalence, and new infections declined from 120,000 in 2010 to 74,000 in 2021 [34]. Despite these advances, gaps persist, particularly in achieving the UNAIDS 95–ID="EN7">95-95 targets, underscoring the need for renewed investments in clinical care to ensure equitable access across populations.

The HIV-CAI was positively associated with both HIV prevalence and HIV incidence, suggesting that higher-burden countries tend to have greater HIV care investment and accessibility. Potential reasons are that targeted investments and intensified treatment efforts are likely to be concentrated in higher-burden countries due to their higher priority, while improved HIV care access may increase documented incidence rates through enhanced case-finding capacities [34]. In contrast, the HIV-CAI demonstrated a negative association with total population, suggesting that more populous countries may encounter scale and distribution challenges that could negatively impact effective access to care. Persistent scale-related barriers in large populations indicate the need for treatment universality programs, international cooperation for equitable global distribution of HIV treatments, and differentiated service delivery through equity-focused subnational strategies [19]. The HIV-CAI provides evidence for construct validity, and its correlation with economic and health-related indicators suggests that the index captures how resource availability influences healthcare access across and within countries. However, further validation studies comparing HIV-CAI with other healthcare access measures and clinical outcomes would strengthen its utility as a tool for assessment.

The differences in HIV clinical care access across geographic areas demonstrate the complexity between healthcare infrastructure, resource allocation, and geographic accessibility in different contexts. While some countries, such as Botswana, Thailand, and Kenya, demonstrated homogeneous access across urban, suburban, and rural areas, suggesting the successful implementation of national HIV care programs, other countries revealed disparities in their healthcare delivery systems.

The unexpected finding that urban areas in Nigeria had the lowest access scores (0.54) compared to suburban areas (0.78) and rural areas (0.59) challenges assumptions about the advantages of urban healthcare and may indicate problems with healthcare system saturation, resource distribution, or HIV care delivery in densely populated urban centers. Similarly, a previous study found that the proportion of individuals in Nigeria experiencing early HIV detection was higher in rural areas than in urban areas [21]. This finding is surprising because rural areas are typically characterized by limited healthcare access and lower health literacy, particularly regarding HIV knowledge [21, 34, 34].

The findings of this study revealed that most HIV medication scores exceeded 0.80, indicating that access to medication and patients' adherence to medication have been successful in reaching diverse populations globally. The consistently high scores observed in Botswana suggest that well-coordinated national HIV programs can effectively ensure medication availability regardless of geographic area. Another study using BAIS V data showed that among PWH living in Botswana who were aware of their status, 98·0% were on ART, and treatment was high in both rural and urban areas [29]. A potential reason for Botswana’s success was the country’s response to the epidemic, which involved improving ART coverage and increasing the number of people who were virally suppressed [29]. This was emphasized in the Third Botswana National Strategic Framework for HIV and AIDS 2019–23, which highlighted the importance of scalable ART by extending free ART to non-citizens [34].

The findings from this study varied, ranging from viral load testing access scores between 0.55 and 1.00, indicating that these disparities appear to be mediated by national healthcare infrastructure and policy. Botswana again led the HIV care access across all geographic areas, exemplifying how national HIV programs can overcome traditional rural–urban healthcare disparities. This finding aligns with previous research documenting Botswana's comprehensive HIV treatment infrastructure [28, 29, 34].

Notably, urban areas do not consistently demonstrate superior access to viral load testing. Nigeria's urban areas recorded the lowest access score in our dataset (0.55). However, the lower rural access in viral load testing was also observed in Nigeria (0.57) and the United States (0.71). These findings are consistent with the public health literature, which documents rural healthcare challenges [21, 34], suggesting the need for increased investment in laboratory infrastructure and point-of-care testing capabilities, especially in rural areas [34]. The disparities identified across and within countries in this study have important implications for HIV treatment outcomes and viral suppression rates. Reduced access to specialized clinics, HIV medication, and viral load testing, particularly in Nigeria's urban areas and rural regions across multiple studied countries, may contribute to delayed treatment, increased HIV transmission, and poorer patient outcomes.

The HIV-CAI’s ability to capture variations across HIV care accessibility domains demonstrates its utility in identifying areas requiring intervention. Moreover, the HIV-CAI established a framework for measuring healthcare accessibility, contributing to the literature and providing a tool for assessing HIV care accessibility across diverse geographic areas and socioeconomic contexts. However, limitations should be considered. First, the study’s convenience sample size varied across countries, which may have affected the reliability of country-level differences. Second, geographic area coverage is limited to individuals who self-reported their HIV care access, which may introduce selection bias and not fully reflect the complexity of each country’s healthcare system. Third, while the study examined some country-level economic and health factors, it may not have captured relevant contextual factors that could influence HIV care access, such as cultural attitudes or specific national healthcare policies. Fourth, part of the data collection occurred during the COVID-19 pandemic, in which health spending may have been redirected to address other urgent health problems related to the pandemic (e.g., immunizations), potentially limiting resources available for HIV care. Fifth, another limitation is the challenge of validating HIV-CAI against established metrics. While we compare index scores between the studied countries and economic and health-related indicators, the need for comparable healthcare access measures in the existing literature restricts our ability to benchmark our findings against those of previous studies. This limitation underscores the need for further validation studies and refinement of comprehensive HIV care access measurements. Sixth, the small sample size of nine countries limited our ability to conduct meaningful comparisons of the mean differences between countries' HIV-CAI scores. Although variations in healthcare access were observed across countries, the sample size was insufficient to determine the statistical significance of these differences at the territorial level. Future studies should involve a larger number of countries. Seventh, the measurements were from a non-validated self-reported survey. Eighth, this study focused on exploring HIV care across geographic locations. In the future, we will use these scores to measure how HIV care access differs across gender identities and sexual orientation. Lastly, strategies to distribute ART during COVID-19 in some countries may have influenced disparities in access to HIV medication. Future research should address the identified limitations by expanding geographic coverage, increasing sample sizes, and incorporating longitudinal data to track changes in HIV care access over time. A more detailed exploration of the structural determinants of health, including health resource appropriations, budgets, and policy interventions [17], such as those aimed at improving rural healthcare infrastructure or expanding telemedicine, is needed to better understand how these structural approaches can help bridge the urban–rural divide for PWH. Despite these limitations, this study advances the public health field by introducing a novel HIV-CAI, which measures the coverage of essential components for improved HIV care, including access to clinics, medication, and viral load monitoring.

## Conclusion

In this study, we developed the HIV-CAI to assess and compare healthcare accessibility across nine countries within urban, suburban, and rural areas. The Index’s ability to capture variations across HIV care accessibility domains, including access to specialized clinics, medication, and viral load monitoring, demonstrates its utility in identifying areas requiring intervention. The findings demonstrate discrepancies in HIV care access within and across countries, highlighting the challenges in achieving equitable and comprehensive HIV care, particularly in rural areas in the pandemic context. Tailored approaches to improving HIV care access are needed to align with UNAIDS’ 95–95–95 target.’ Increasing the proportion of PWH with access to HIV treatment will assist in achieving this target. The urban–rural failure in care, especially access to HIV clinical care and HIV care providers and viral load testing, calls for targeted interventions to bridge this gap and move closer to the goal of ending the HIV epidemic and ensuring better health outcomes for all individuals living with HIV.

## Supplementary Information

Below is the link to the electronic supplementary material.

ESM 1 (DOCX 111 KB)

## Data Availability

The public data used in these analyses can be downloaded from the World Bank Group, World Development Indicators website at https://databank.worldbank.org/source/world-development-indicators. The datasets generated and/or analyzed from the International Nursing Network for HIV Research Study VIII are not publicly available due to the sensitive health information (e.g., HIV status) of the participants, but are available from the corresponding author upon reasonable request.

## References

[1] UNAIDS. UNAIDS Data 2021. 2021. https://www.unaids.org/en/resources/documents/2021/2021_unaids_data. Accessed 17 Jul 2024

[2] Joint United Nations Programme on HIV/AIDS. Fast Track: Ending the AIDS Epidemic by 2030. Geneva, Switzerland: United Nations, Economic and Social Council, Joint United Nations Programme on HIV/AIDS.; 2014. https://www.unaids.org/sites/default/files/media_asset/JC2686_WAD2014report_en.pdf. Accessed 17 Jul 2024.

[3] UNAIDS. Understanding Fast-Track: Accelerating Action to End the AIDS Epidemic by 2030; 2015. https://www.unaids.org/sites/default/files/media_asset/201506_JC2743_Understanding_FastTrack_en.pdf. Accessed 17 Jul 2024.

[4] UNAIDS. 2025 AIDS Target. Putting People Living with HIV and Communities at Risk at the Centre. https://aidstargets2025.unaids.org/#section-targets. Accessed 6 Dec 2024.

[5] Frank TD, Carter A, Jahagirdar D, et al. Global, regional, and national incidence, prevalence, and mortality of HIV, 1980–2017, and forecasts to 2030, for 195 countries and territories: a systematic analysis for the global burden of diseases, injuries, and risk factors study 2017. Lancet HIV. 2019;6(12):e831–59. 10.1016/S2352-3018(19)30196-1. 31439534 10.1016/S2352-3018(19)30196-1 PMC6934077

[6] UNAIDS. Global HIV & AIDS Statistics — Fact Sheet.; 2023.https://www.unaids.org/en/resources/fact-sheet Accessed 15 Oct 2024

[7] Gallant JE, Adimora AA, Carmichael JK, et al. Essential components of effective HIV care: a policy paper of the HIV medicine association of the infectious diseases society of America and the Ryan White medical providers coalition. Clin Infect Dis. 2011;53(11):1043–50. 10.1093/cid/cir689. 22021928 10.1093/cid/cir689 PMC3205204

[8] Person AK, Armstrong WS, Evans T, et al. Principles for ending human immunodeficiency virus as an epidemic in the United States: a policy paper of the Infectious Diseases Society of America and the HIV Medicine Association. Clin Infect Dis. 2023;76(1):1–9. 10.1093/cid/ciac626. 35965395 10.1093/cid/ciac626

[9] Cohen MS, Chen YQ, McCauley M, et al. Prevention of HIV-1 infection with early antiretroviral therapy. N Engl J Med. 2011;365(6):493–505. 10.1056/NEJMoa1105243. 21767103 10.1056/NEJMoa1105243 PMC3200068

[10] Gandhi RT, Bedimo R, Hoy JF, et al. Antiretroviral drugs for treatment and prevention of HIV infection in adults: 2022 recommendations of the International Antiviral Society–USA panel. JAMA. 2023;329(1):63. 10.1001/jama.2022.22246. 36454551 10.1001/jama.2022.22246

[11] Endalamaw A, Gilks CF, Ambaw F, Habtewold TD, Assefa Y. Universal health coverage for antiretroviral treatment: a review. Infect Dis Rep. 2022;15(1):1–15. 10.3390/idr15010001. 36648855 10.3390/idr15010001 PMC9844463

[12] Gaolathe T, Wirth KE, Holme MP, et al. Botswana’s progress toward achieving the 2020 UNAIDS 90–90-90 antiretroviral therapy and virological suppression goals: a population-based survey. Lancet HIV. 2016;3(5):e221–30. 10.1016/S2352-3018(16)00037-0. 27126489 10.1016/S2352-3018(16)00037-0 PMC5146754

[13] Oturu K, O’Brien O, Ozo-Eson PI. Barriers and enabling structural forces affecting access to antiretroviral therapy in Nigeria. BMC Public Health. 2024;24(1):105. 10.1186/s12889-023-17271-6. 38184516 10.1186/s12889-023-17271-6 PMC10770989

[14] Aung NHHL, Soe KT, Kumar AMV, Saw S, Aung ST. What are the barriers for uptake of antiretroviral therapy in HIV-infected tuberculosis patients? A mixed-methods study from Ayeyawady region, Myanmar. TropicalMed. 2020;5(1): 41. 10.3390/tropicalmed5010041. 10.3390/tropicalmed5010041 PMC7157433 32182967

[15] Fauk NK, Gesesew HA, Seran AL, Raymond C, Tahir R, Ward PR. Barriers to accessing HIV care services in host low and middle income countries: views and experiences of Indonesian male ex-migrant workers living with HIV. Int J Environ Res Public Health. 2022;19(21): 14377. 10.3390/ijerph192114377. 36361253 10.3390/ijerph192114377 PMC9654942

[16] Waterfield KC, Shah GH, Etheredge GD, Ikhile O. Consequences of COVID-19 crisis for persons with HIV: the impact of social determinants of health. BMC Public Health. 2021;21(1):299. 10.1186/s12889-021-10296-9. 33546659 10.1186/s12889-021-10296-9 PMC7863613

[17] Heller JC, Givens ML, Johnson SP, Kindig DA. Keeping it political and powerful: defining the structural determinants of health. Milbank Q. 2024;102(2):351–66. 10.1111/1468-0009.12695. 38363858 10.1111/1468-0009.12695 PMC11176401

[18] Cuca YP, Horvat Davey C, Corless IB, et al. The social, mental, and physical health impacts of the COVID-19 pandemic on people with HIV: protocol of an observational international multisite study. J Assoc Nurses AIDS Care. 2024;35(1):60–74. 10.1097/JNC.0000000000000444. 38096186 10.1097/JNC.0000000000000444 PMC10749681

[19] Bouabida K, Chaves BG, Anane E. Challenges and barriers to HIV care engagement and care cascade: viewpoint. Front Reprod Health. 2023;5: 1201087. 10.3389/frph.2023.1201087. 37547803 10.3389/frph.2023.1201087 PMC10398380

[20] Nelson JA, Kinder A, Johnson AS, et al. Differences in selected HIV care continuum outcomes among people residing in rural, urban, and metropolitan areas—28 US jurisdictions. J Rural Health. 2018;34(1):63–70. 10.1111/jrh.12208. 27620836 10.1111/jrh.12208

[21] Lawal TV, Oyedele OK, Andrew NP. On characterizing gender and locational composition of adult PLHIV in Nigeria: implications for HIV programming. PLOS Glob Public Health. 2024;4(8):e0002863. 10.1371/journal.pgph.0002863. Robinson J, editor. 10.1371/journal.pgph.0002863 PMC11346663 39186499

[22] HIV.gov. HIV Care Continuum.; 2022. https://www.hiv.gov/federal-response/policies-issues/hiv-aids-care-continuum. Accessed 9 Jul 2024

[23] Kay ES, Batey DS, Mugavero MJ. The HIV treatment cascade and care continuum: updates, goals, and recommendations for the future. AIDS Res Ther. 2016;13(1):35. 10.1186/s12981-016-0120-0. 27826353 10.1186/s12981-016-0120-0 PMC5100316

[24] Association of Nurses in AIDS Care. *International Nursing Network for HIV/AIDS Research*. Accessed July 9, 2024. https://www.nursesinaidscare.org/i4a/pages/index.cfm?pageid=4551 10.1097/JNC.0000000000000273 33929982

[25] Dicker D, Nguyen G, Abate D, et al. Global, regional, and national age-sex-specific mortality and life expectancy, 1950–2017: a systematic analysis for the Global Burden of Disease Study 2017. Lancet. 2018;392(10159):1684–735. 10.1016/S0140-6736(18)31891-9. 30496102 10.1016/S0140-6736(18)31891-9 PMC6227504

[26] Lopez-Rios J. Missed opportunities for HIV prevention in Puerto Rico: an argument for inclusivity and community coalitions. Am J Public Health. 2021;111(7):1265–6. 10.2105/AJPH.2021.306342. 34110935 10.2105/AJPH.2021.306342 PMC8493179

[27] World Bank Groups. World Development Indicators. https://databank.worldbank.org/source/world-development-indicators. Accessed 9 July 2024.

[28] Ramogola-Masire D, Poku O, Mazhani L, et al. Botswana’s HIV response: policies, context, and future directions. J Community Psychol. 2020;48(3):1066–70. 10.1002/jcop.22316. 31951283 10.1002/jcop.22316 PMC7103557

[29] Mine M, Stafford KA, Laws RL, et al. Progress towards the UNAIDS 95–95-95 targets in the fifth Botswana AIDS impact survey (BAIS V 2021): a nationally representative survey. Lancet HIV. 2024;11(4):e245–54. 10.1016/S2352-3018(24)00003-1. 38467135 10.1016/S2352-3018(24)00003-1 PMC11289895

[30] Siraprapasiri T, Ongwangdee S, Benjarattanaporn P, Peerapatanapokin W, Sharma M. The impact of Thailand’s public health response to the HIV epidemic 1984–2015: understanding the ingredients of success. J Virus Eradication. 2016;2:7–14. 10.1016/S2055-6640(20)31093-1. 10.1016/S2055-6640(20)31093-1 PMC5337415 28275444

[31] Salihu A, Jahun I, Oyedeji DO, et al. Scaling up access to antiretroviral treatment for HIV: lessons from a key populations program in Nigeria. AIDS Res Ther. 2025;22(1):10. 10.1186/s12981-025-00711-1. 39893486 10.1186/s12981-025-00711-1 PMC11787728

[32] Onovo AA, Adeyemi A, Onime D, et al. Estimation of HIV prevalence and burden in Nigeria: a Bayesian predictive modelling study. eClinicalMedicine. 2023;62:102098. 10.1016/j.eclinm.2023.102098 10.1016/j.eclinm.2023.102098 PMC10393599 37538543

[33] United Nations Nigeria. *From the Darkest of Days to a New Dawn: 35 Years of the Nigerian Response to HIV and AIDS*. UNAIDS; 2022.

[34] Carter A, Zhang M, Tram KH, et al. Global, regional, and national burden of HIV/AIDS, 1990–2021, and forecasts to 2050, for 204 countries and territories: the global burden of disease study 2021. Lancet HIV. 2024;11(12):e807–22. 10.1016/S2352-3018(24)00212-1. 39608393 10.1016/S2352-3018(24)00212-1 PMC11612058

[35] Owens C, Voorheis E, Lester JN, et al. The lived experiences of rural HIV social workers. AIDS Care. 2023;35(1):48–52. 10.1080/09540121.2021.1981817. 34612112 10.1080/09540121.2021.1981817

[36] Dos Santos FC, Garofalo R, Kuhns LM, Scherr T, Schnall R. Evaluating the effectiveness of a mobile health intervention on enhancing HIV knowledge in sexual and gender minority men. JAIDS J Acquir Immune Defic Syndr. 2025;98(3):217–23. 10.1097/QAI.0000000000003562. 39813261 10.1097/QAI.0000000000003562 PMC12179779

[37] National AIDS and Health Promotion Agency. *The Third Botswana National Strategic Framework for HIV & AIDS, 2019–2023*; 2019.

[38] Jefferis K, Avalos A, Phillips H, et al. Five years after treat all implementation: botswana’s HIV response and future directions in the era of COVID-19. S Afr J HIV Med. 2021. 10.4102/sajhivmed.v22i1.1275. 10.4102/sajhivmed.v22i1.1275 PMC8602965 34853703

[39] Schafer KR, Albrecht H, Dillingham R, et al. The continuum of HIV care in rural communities in the United States and Canada: what is known and future research directions. JAIDS J Acquir Immune Defic Syndr. 2017;75(1):35–44. 10.1097/QAI.0000000000001329. 28225437 10.1097/QAI.0000000000001329 PMC6169533

[40] Ochodo EA, Olwanda EE, Deeks JJ, Mallett S. Point-of-care viral load tests to detect high HIV viral load in people living with HIV/AIDS attending health facilities. Cochrane Infectious Diseases Group, editor. Cochrane Database of Systematic Reviews. 2022;2022(3). 10.1002/14651858.CD013208.pub2 10.1002/14651858.CD013208.pub2 PMC8908762 35266555

