# Supplementary material for: Global Measures of HIV Care Accessibility Across Urban, Suburban, and Rural Areas
Source: J Urban Health. 2025 Dec 8;102(6):1208–22. doi: 10.1007/s11524-025-01021-7 (PMC12738474; doi:10.1007/s11524-025-01021-7)
Supplement: Supplementary file 1 — (DOCX 111 KB) [file 11524_2025_1021_MOESM1_ESM.docx]

**Supplemental Online Content**

**Global Measures of HIV Care Accessibility across Urban, Suburban, and Rural Areas**

Fabiana Dos Santos, PhD, MSN, RN^1^, Panta Apiruknapanond, PhD, RN,^2^ Tongyao Wang, PhD, RN,^3^ Carol Dawson-Rose, PhD, RN, FAAN,^4^ Claudia P. Valencia- Molina, PhD, RN^5^, Christine Horvat Davey, PhD, RN,^6^ Solymar Solís Báez, BA,^7^ Emilia Iwu, PhD, RN,^8^ Motshedisi Sabone, PhD, RN,^9^ Lufuno Makhado, PhD, RN,^10^ J. Craig Phillips, PhD, LLM, RN, ACRN, FAAN, FCAN,^11^ Inge B. Corless, PhD, MA, BSN, FNAP, FAAN,^12^ Sheila Shaibu, PhD, RN,^13^ Wei-Ti Chen, PhD, RN, CNM, FAAN,^14^ Diane Santa Maria, DrPH, RN, PHNA-BC, FASHM, FAAN,^15^ Yvette P. Cuca, PhD, MPH, MIA,^4^ Rebecca Schnall, PhD, MPH, RN, FAAN, FACMI^16^

^1^Purdue University School of Nursing, West Lafayette, Indiana, USA

^2^ Chulalongkorn University, St. Louis College, School of Nursing, Bangkok, Thailand

^3^The University of Hong Kong, School of Nursing, Pokfulam, Hong Kong

^4^University of California, School of Nursing, Department of Community Health Systems, San Francisco, California, USA

^5^Universidad del Valle, Escuela de Enfermería, Facultad de Salud, Cali, Colombia

^6^Case Western Reserve University, Frances Payne Bolton School of Nursing, Cleveland, Ohio, USA

^7^Auxilio Mutuo Hospital, San Juan, Puerto Rico

^8^Rutgers University, School of Nursing, Center for Global Health, and Senior Technical Advisor, Institute of Human Virology, Newark, New Jersey, USA

^9^Retired Professor of Nursing, Gaborone, Botswana

^10^University of Venda, Department of Public Health, Limpopo Province, South Africa

^11^ University of Ottawa, Faculty of Health Sciences, School of Nursing, Ottawa, Canada

^12^Professor Emerita, Massachusetts General Hospital Institute of Health Professions School of Nursing, Boston, Massachusetts, USA

^13^Aga Khan University, School of Nursing, Nairobi, Kenya

^14^University of California, Los Angeles, Joe C. Wen School of Nursing, Los Angeles, California, USA

^15^University of Texas Health Science Center at Houston, Cizik School of Nursing, Houston, Texas, USA

^16^Columbia University, School of Nursing, New York, USA

**Corresponding author:** Fabiana Cristina Dos Santos, PhD, MSN, RN. Assistant Professor

Purdue University School of Nursing. 610 Purdue Mall, West Lafayette, IN, 47906. Email address: fdossant@purdue.edu. ORCID: [0000-0001-9780-4336](https://orcid.org/0000-0001-9780-4336)

**List of contents**

**eTable 1.** World Development Indicators.

**eTable 2.** Original Survey Variables for HIV Care Access by Domains.

**eTable 3.** Normalized HIV Care Access Variables.

**eTable 4.** HIV Care Access Index Scores by Country with Distributional Percentiles.

**eFigure 1.** Scatterplot illustrates the relationship between economic and health determinants and HIV care access.

This supplemental material has been provided by the authors to give readers additional information about this work.

**eTable 1.** World Development Indicators

| Indicators^a^ | Descriptions |
| --- | --- |
| Gross Domestic Product - GDP (constant 2015 US$) | Measures of economic activity and the value of a country's final goods and services, including product taxes and excluding subsidies not factored into product prices |
| Current Health Expenditure (% of GDP) | Indicates the proportion of a country’s economic resources directed towards health, reflecting the health sector’s importance in the overall economy |
| Incidence of HIV, (per 1,000 uninfected population) | Number of new HIV infections among 1,000 uninfected population in the year preceding the measurement period |
| Prevalence of HIV, total (% of the population ages 15-49) | The percentage of people ages 15-49 who are infected with HIV |
| ART Coverage (% of people living with HIV) | Indicates the percentage of all people living with HIV who are receiving ART |
| Total Population | Refers to the count of all residents in a country, regardless of their documented status or citizenship |
| Poverty Headcount Ratio at the Societal Poverty Line (% of the population) | Refers to the percentage of a population living in poverty according to the World Bank's societal poverty line |
| Prevalence of severe food insecurity in the population (%) | The percentage of people living in households classified as severely food insecure |
| Current Health Expenditure Per Capita, PPP (current international $) | The average health spending per person in a standardized currency, accounting for the purchasing power of each country's currency compared to the US dollar, which helps compare health expenditures across different countries relative to population size |

^a^Indicators from the database World Development Indicators [Last Updated: 03/28/2024].

**eTable 2.** Original Survey Variables for HIV Care Access by Domains

| Domain 1. Access to HIV Clinical Care and HIV Care Providers | | Domain 2. Access to HIV Medication | | Domain 3. Access to Viral Load Testing | |
| --- | --- | --- | --- | --- | --- |
| Variable | Response Option | Variable | Response Option | Variable | Response Option |
| 1. In the past 12 months, how many times have you seen your HIV healthcare provider? | Ranging from Never (0) to >12 visits (13) | 6. Have you taken antiretroviral (ARV) medication? | I have never taken ARV medication (1), I am currently taking ARV (2), I started taking ARV but have since stopped taking it (3) | 11. What was your most recent viral load? | Undetectable (1),  Detectable (2) |
| 2. Have you had trouble making or keeping your HIV care appointments with your doctor because of COVID-19 or the public health efforts to manage it? | No (0), Yes (1), I have not tried to have an appointment (2) | 7. Have you had trouble getting access to HIV medications? | Ranging from Has highly decreased because of COVID-19 (1) to Has highly increased because of COVID-19 (5) | 12. Have you had trouble getting viral loads or other labs done?^b^ | Ranging from Has highly decreased because of COVID-19 (1) to Has highly increased because of COVID-19 (5) |
| 3. Have you had trouble getting HIV care clinical visits? | Ranging from Has highly decreased because of COVID-19 (1) to Has highly increased because of COVID-19 (5) | 8. Have you had trouble getting daily adherence to HIV medications? | Ranging from Has highly decreased because of COVID-19 (1) to Has highly increased because of COVID-19 (5) |  |  |
| 4. Have you had trouble getting your HIV medication prescriptions from your doctor because of COVID-19 or the public health efforts to manage it? | No (0), Yes (1), I have not tried to have an appointment (2) | 9. For the past 3 days, what percentage of the time were you able to take your HIV medications exactly as prescribed? | Ranging from (0) to (100) |  |  |
| 5. Have you had trouble getting your HIV medication prescriptions filled at the pharmacy because of COVID-19 or the public health efforts to manage it?^a^ | No (0), Yes (1), I have not tried to have an appointment (2) | 10. For the past 30 days, what percentage of the time were you able to take your HIV medications exactly as prescribed? | Ranging from (0) to (100) |  |  |

1. Domain 1/item 5 categorization reflects pharmacy-related medication access. b. Domain 3/item 12 categorization prioritizes viral load testing.

**eTable 3.** Normalized HIV Care Access Variables

| Domain 1. Access to Clinical Care and Health Care Provider | | Domain 2. Access to HIV Medication | | Domain 3. Access to Viral Load Testing | |
| --- | --- | --- | --- | --- | --- |
| Variable | Response Option | Variable | Response Option | Variable | Response Option |
| In the past 12 months, how many times have you seen your HIV healthcare provider? | Decimals between 0 and 1 | Have you taken antiretroviral (ARV) medication? | I have never taken ARV medication (0) | What was your most recent viral load? | Detectable (1)/Undetectable (0) |
|  |  |  | I am currently taking ARV medication (1) |  |  |
| Have you had trouble making or keeping your HIV care appointments with your doctor because of COVID-19 or the public health efforts to manage it? | Yes (1)/No (0) | Have you had trouble getting access to HIV medications? | Reduced access to HIV medications (0) | Have you had trouble getting viral loads or other labs done? | Reduced access to viral load testing (0) |
|  |  |  |  |  | Increased access to viral load testing (1) |
|  |  |  | Increased access to HIV medications (1) |  |  |
| Have you had trouble getting HIV care clinical visits? | Reduced clinical visits (0) | Have you had trouble getting daily adherence to HIV medications? | Reduced daily adherence to HIV medication (0) |  |  |
|  | Increased clinical visits (1) |  |  |  |  |
|  |  |  | Increased daily adherence to HIV medication (1) |  |  |
| Have you had trouble getting your HIV medication prescriptions from your doctor because of COVID-19 or the public health efforts to manage it? | Yes (1)/No (0) | For the past 3 days, what percentage of the time were you able to take your HIV medications exactly as prescribed? | Decimals between 0 and 1 |  |  |
| Have you had trouble getting your HIV medication prescriptions filled at the pharmacy because of COVID-19 or the public health efforts to manage it? | Yes (1)/No (0) | For the past 30 days, what percentage of the time were you able to take your HIV medications exactly as prescribed? | Decimals between 0 and 1 |  |  |

**eTable 4.** HIV Care Access Index Scores by Country with Distributional Percentiles

| Country | Sample Size  (n = 1,598) | HIV Care Access Index (Average) | HIV Care Access Index | | |
| --- | --- | --- | --- | --- | --- |
|  |  |  | 0.25 Percentile | 0.50 Percentile | 0.75 Percentile |
| Botswana | 100 | 0.93 | 0.95 | 0.96 | 0.96 |
| Colombia | 100 | 0.83 | 0.90 | 0.96 | 0.97 |
| China | 270 | 0.79 | 0.66 | 1.00 | 1.00 |
| Kenya | 112 | 0.90 | 0.90 | 0.96 | 0.97 |
| Nigeria | 119 | 0.64 | 0.21 | 0.78 | 0.96 |
| Puerto Rico | 23 | 0.88 | 0.96 | 0.96 | 0.96 |
| South Africa | 285 | 0.88 | 0.89 | 0.97 | 1.00 |
| Thailand | 104 | 0.91 | 0.95 | 0.96 | 0.96 |
| United States | 485 | 0.81 | 0.77 | 0.96 | 0.97 |


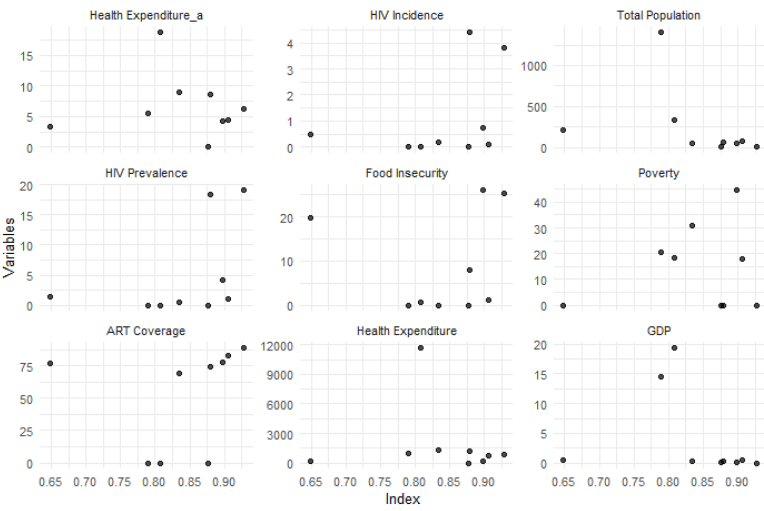


**eFigure 1.** Scatterplot illustrates the relationship between economic and health determinants and HIV care access.
